# 2-(Dibutyl­amino)-3-(4-fluoro­phen­yl)-5,6,7,8-tetra­hydro-7-methyl-6,8-di­phenyl­pyridine­[3′,4′:2,3]thieno[5,4-*d*]pyrimidin-4(3*H*)-one

**DOI:** 10.1107/S1600536808002845

**Published:** 2008-01-30

**Authors:** Guo-ping Zeng, Qing Li, Yang-gen Hu

**Affiliations:** a Department of Chemistry and Life Science, Hubei University of Education, Wuhan 430205, People’s Republic of China; b Taihe Hospital, Yunyang Medical College, Shiyan 442000, People’s Republic of China; c Department of Medicinal Chemistry, Yunyang Medical College, Shiyan 442000, People’s Republic of China

## Abstract

In the crystal structure of the title compound, C_36_H_39_FN_4_OS, the two fused rings of the thienopyrimidine system are coplanar. The 4-fluoro­phenyl ring is twisted with respect to the heterocyclic pyrimidinone ring by 67.21 (14)°. The piperidine ring shows a half-chair conformation. One of the *n*-butyl chains is disordered equally over two sites. The crystal packing is stabilized by C—H⋯O hydrogen bonds.

## Related literature

The preparation and biological activity are described by Walter (1999*a* ▶,*b* ▶). For related literature, see: Ding *et al.* (2004 ▶). For the crystal structures of other fused pyrimidinone derivatives, see: Hu *et al.* (2006 ▶, 2007 ▶).
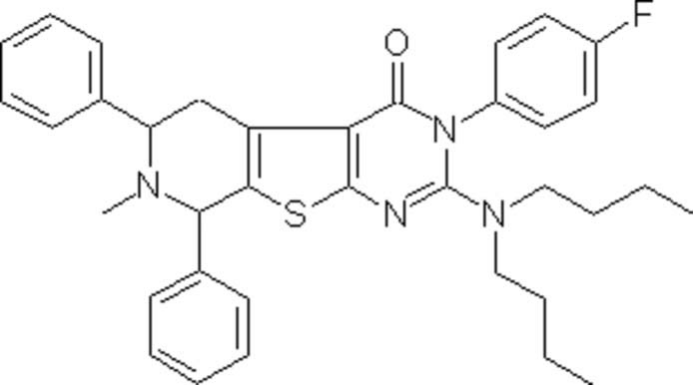


## Experimental

### 

#### Crystal data

C_36_H_39_FN_4_OS
*M* *_r_* = 594.77
Monoclinic, 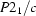

*a* = 13.723 (4) Å
*b* = 9.836 (3) Å
*c* = 24.5496 (15) Å
β = 101.342 (2)°
*V* = 3249.0 (14) Å^3^
*Z* = 4
Mo *K*α radiation
μ = 0.14 mm^−1^
*T* = 294 (2) K
0.20 × 0.10 × 0.10 mm

#### Data collection

Bruker SMART 4K CCD area-detector diffractometer
Absorption correction: multi-scan (*SADABS*; Bruker, 2001 ▶) *T*_min_ = 0.973, *T*_max_ = 0.986
32225 measured reflections
6359 independent reflections
4457 reflections with *I* > 2σ(*I*)
*R*_int_ = 0.049

#### Refinement

*R*[*F* ^2^ > 2σ(*F* ^2^)] = 0.075
*wR*(*F* ^2^) = 0.178
*S* = 1.10
6359 reflections
420 parameters
H-atom parameters constrained
Δρ_max_ = 0.40 e Å^−3^
Δρ_min_ = −0.18 e Å^−3^

### 

Data collection: *SMART* (Bruker, 2001 ▶); cell refinement: *SAINT-Plus* (Bruker, 2001 ▶); data reduction: *SAINT-Plus*; program(s) used to solve structure: *SHELXS97* (Sheldrick, 2008 ▶); program(s) used to refine structure: *SHELXL97* (Sheldrick, 2008 ▶); molecular graphics: *PLATON* (Spek, 2003 ▶); software used to prepare material for publication: *SHELXTL* (Sheldrick, 2008 ▶).

## Supplementary Material

Crystal structure: contains datablocks I, global. DOI: 10.1107/S1600536808002845/bt2671sup1.cif

Structure factors: contains datablocks I. DOI: 10.1107/S1600536808002845/bt2671Isup2.hkl

Additional supplementary materials:  crystallographic information; 3D view; checkCIF report

## Figures and Tables

**Table 1:** Hydrogen-bond geometry (Å, °)

*D*—H⋯*A*	*D*—H	H⋯*A*	*D*⋯*A*	*D*—H⋯*A*
C4—H4⋯O1^i^	0.93	2.47	3.304 (6)	149

Symmetry code: (i) 
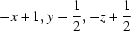
.

**Fig. 1.**
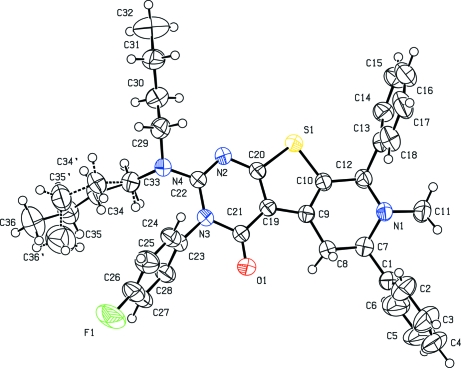
Molecular structure of the title compound with labelling and displacement ellipsoids drawn at the 50% probability level

## supplementary crystallographic information

### Comment

The derivatives of heterocycles containing the thienopyrimidine system, which are well known bioisosteres of quinazolines, are of great importance because of their remarkable biological properties (Walter, 1999*a*; Walter, 1999*b*; Ding *et al.*, 2004). Recently, we have focused on the synthesis of the fused heterocycle systems containing thienopyrimidine *via* aza-Wittig reaction at room temperature. Some X-ray crystal structures of fused pyrimidinone derivatives have been reported (Hu *et al.*, 2006; 2007). The title compound (Fig. 1) may be used as a precursor for obtaining bioactive molecules. The two fused rings of the thienopyrimidine ring system are coplanar, making a dihedral angle of 0.36 (13)°. The *p*-fluorophenyl ring is twisted with respect to pyrimidinone ring by 67.21 (14)°. The piperidine ring shows a half-chair conformation [φ =25.9 (4)° and θ = 49.9 (3)°, puckering Amplitude = 0.515 (3) Å]. One of the *n*-butyl chains is disordered over two sites. The crystal packing is stabilized by C—H···O hydrogen bonds interactions (Table 1).

### Experimental

To a solution of ethyl 2-((*p*-flurophenylimino)methyleneamino)-4,5,6,7-tetrahydro-6-methyl- 5,7-diphenylthieno[2,3-*c*]pyridine-3-carboxylate (3 mmol) in dichloromethane (5 ml) was added dibutylamine (3 mmol). After stirring the reaction mixture for 1 h, the solvent was removed and anhydrous ethanol (10 ml) with several drops of EtONa in EtOH was added. The mixture was stirred for 4 h at room temperature. The solution was concentrated under reduced pressure and the residue was recrystallized from dichloromethane and ethanol (*v*/*v* = 1:1) to give the title compound in a yield of 80%. Crystals suitable for single-crystal X-ray diffraction were obtained by recrystallization from a mixed solvent of ethanol and dichloromethane (1:3 *v*/*v*) at room temperature.

### Refinement

All H-atoms were positioned with idealized geometry and refined using a riding model with *U*_iso_(H)= 1.5*U*_eq_(C) for methyl H atoms and *U*_iso_(H) =1.2*U*_eq_(C) for all other H atoms and with C—H ranging from 0.93° to 0.98 Å. The methyl groups were allowed to rotate but not to tip. Three atoms of one *n*-butyl chains are disordered over two sites with site occupation factors of 0.540 (12) and 0.460 (12).

### Crystal data

**Table:**

C_36_H_39_FN_4_OS	*F*_000_ = 1264
*M**_r_* = 594.77	*D*_x_ = 1.216 Mg m^−^^3^
Monoclinic, *P*2_1_/*c*	Mo *K*α radiation λ = 0.71073 Å
Hall symbol: -P 2ybc	Cell parameters from 4792 reflections
*a* = 13.723 (4) Å	θ = 2.2–21.1º
*b* = 9.836 (3) Å	µ = 0.14 mm^−^^1^
*c* = 24.5496 (15) Å	*T* = 294 (2) K
β = 101.342 (2)º	Block, colorless
*V* = 3249.0 (14) Å^3^	0.20 × 0.10 × 0.10 mm
*Z* = 4	

### Data collection

**Table:**

Bruker SMART 4K CCD area-detector diffractometer	6359 independent reflections
Radiation source: fine-focus sealed tube	4457 reflections with *I* > 2σ(*I*)
Monochromator: graphite	*R*_int_ = 0.049
*T* = 294(2) K	θ_max_ = 26.0º
φ and ω scans	θ_min_ = 2.0º
Absorption correction: multi-scan(SADABS; Bruker, 2001)	*h* = −16→16
*T*_min_ = 0.973, *T*_max_ = 0.986	*k* = −12→12
32225 measured reflections	*l* = −29→30

### Refinement

**Table:**

Refinement on *F*^2^	Secondary atom site location: difference Fourier map
Least-squares matrix: full	Hydrogen site location: inferred from neighbouring sites
*R*[*F*^2^ > 2σ(*F*^2^)] = 0.075	H-atom parameters constrained
*wR*(*F*^2^) = 0.178	*w* = 1/[σ^2^(*F*_o_^2^) + (0.0743*P*)^2^ + 1.2986*P*] where *P* = (*F*_o_^2^ + 2*F*_c_^2^)/3
*S* = 1.10	(Δ/σ)_max_ < 0.001
6359 reflections	Δρ_max_ = 0.40 e Å^−^^3^
420 parameters	Δρ_min_ = −0.18 e Å^−^^3^
Primary atom site location: structure-invariant direct methods	Extinction correction: none

### Special details

**Table:**

Geometry. All e.s.d.'s (except the e.s.d. in the dihedral angle between two l.s. planes) are estimated using the full covariance matrix. The cell e.s.d.'s are taken into account individually in the estimation of e.s.d.'s in distances, angles and torsion angles; correlations between e.s.d.'s in cell parameters are only used when they are defined by crystal symmetry. An approximate (isotropic) treatment of cell e.s.d.'s is used for estimating e.s.d.'s involving l.s. planes.
Refinement. Refinement of *F*^2^ against ALL reflections. The weighted *R*-factor *wR* and goodness of fit *S* are based on *F*^2^, conventional *R*-factors *R* are based on *F*, with *F* set to zero for negative *F*^2^. The threshold expression of *F*^2^ > σ(*F*^2^) is used only for calculating *R*-factors(gt) *etc*. and is not relevant to the choice of reflections for refinement. *R*-factors based on *F*^2^ are statistically about twice as large as those based on *F*, and *R*- factors based on ALL data will be even larger.

### Fractional atomic coordinates and isotropic or equivalent isotropic displacement parameters (Å^2^)

**Table:**

	*x*	*y*	*z*	*U*_iso_*/*U*_eq_	Occ. (<1)
C1	0.3447 (2)	0.2687 (3)	0.22128 (13)	0.0579 (8)	
C2	0.3770 (3)	0.3694 (5)	0.19044 (17)	0.0870 (12)	
H2	0.3466	0.4543	0.1877	0.104*	
C3	0.4545 (4)	0.3456 (7)	0.1635 (2)	0.1172 (18)	
H3	0.4759	0.4152	0.1431	0.141*	
C4	0.4996 (4)	0.2237 (9)	0.1661 (3)	0.136 (3)	
H4	0.5511	0.2084	0.1472	0.163*	
C5	0.4692 (4)	0.1243 (7)	0.1963 (3)	0.131 (2)	
H5	0.5001	0.0399	0.1982	0.158*	
C6	0.3933 (3)	0.1448 (4)	0.22453 (19)	0.0905 (13)	
H6	0.3745	0.0750	0.2459	0.109*	
C7	0.2588 (2)	0.2901 (3)	0.25038 (12)	0.0510 (7)	
H7	0.2445	0.2034	0.2668	0.061*	
C8	0.2858 (2)	0.3941 (3)	0.29718 (11)	0.0510 (7)	
H8A	0.3355	0.3555	0.3267	0.061*	
H8B	0.3143	0.4739	0.2832	0.061*	
C9	0.19730 (18)	0.4348 (3)	0.31993 (10)	0.0411 (6)	
C10	0.10476 (18)	0.4041 (3)	0.29181 (11)	0.0427 (6)	
C11	0.1498 (2)	0.2452 (4)	0.16164 (14)	0.0767 (11)	
H11A	0.1524	0.1519	0.1733	0.115*	
H11B	0.1998	0.2612	0.1400	0.115*	
H11C	0.0854	0.2645	0.1396	0.115*	
C12	0.08097 (19)	0.3292 (3)	0.23755 (11)	0.0474 (7)	
H12	0.0669	0.2341	0.2450	0.057*	
C13	−0.0112 (2)	0.3913 (3)	0.20114 (11)	0.0503 (7)	
C14	−0.1018 (2)	0.3286 (4)	0.19700 (14)	0.0644 (9)	
H14	−0.1059	0.2453	0.2143	0.077*	
C15	−0.1881 (3)	0.3895 (5)	0.16690 (18)	0.0912 (13)	
H15	−0.2494	0.3472	0.1643	0.109*	
C16	−0.1813 (3)	0.5119 (5)	0.14142 (17)	0.0936 (14)	
H16	−0.2383	0.5533	0.1217	0.112*	
C17	−0.0902 (3)	0.5741 (4)	0.14485 (14)	0.0851 (12)	
H17	−0.0860	0.6567	0.1270	0.102*	
C18	−0.0060 (3)	0.5145 (4)	0.17451 (13)	0.0715 (10)	
H18	0.0552	0.5571	0.1768	0.086*	
C19	0.19517 (18)	0.5079 (3)	0.37045 (10)	0.0398 (6)	
C20	0.10039 (18)	0.5317 (3)	0.37838 (10)	0.0421 (6)	
C21	0.27613 (19)	0.5607 (3)	0.41048 (11)	0.0416 (6)	
C22	0.14679 (18)	0.6441 (3)	0.45949 (11)	0.0438 (7)	
C23	0.32616 (19)	0.6976 (3)	0.49318 (11)	0.0434 (6)	
C24	0.3317 (2)	0.8363 (3)	0.49281 (12)	0.0539 (8)	
H24	0.2826	0.8865	0.4699	0.065*	
C25	0.4101 (2)	0.9018 (4)	0.52640 (15)	0.0714 (10)	
H25	0.4140	0.9962	0.5271	0.086*	
C26	0.4817 (2)	0.8244 (4)	0.55861 (15)	0.0740 (10)	
C27	0.4788 (2)	0.6871 (4)	0.55940 (14)	0.0716 (10)	
H27	0.5289	0.6376	0.5819	0.086*	
C28	0.3998 (2)	0.6217 (3)	0.52609 (12)	0.0581 (8)	
H28	0.3962	0.5273	0.5258	0.070*	
C29	0.0333 (2)	0.7957 (3)	0.49500 (14)	0.0594 (8)	
H29A	0.0426	0.8720	0.5205	0.071*	
H29B	0.0223	0.8328	0.4577	0.071*	
C30	−0.0583 (2)	0.7227 (4)	0.50169 (15)	0.0665 (9)	
H30A	−0.0484	0.6841	0.5387	0.080*	
H30B	−0.0704	0.6484	0.4752	0.080*	
C31	−0.1489 (2)	0.8153 (4)	0.49285 (15)	0.0700 (10)	
H31A	−0.1369	0.8882	0.5199	0.084*	
H31B	−0.1571	0.8559	0.4562	0.084*	
C32	−0.2429 (3)	0.7447 (5)	0.4977 (2)	0.1140 (17)	
H32A	−0.2572	0.6750	0.4699	0.171*	
H32B	−0.2965	0.8092	0.4925	0.171*	
H32C	−0.2357	0.7043	0.5339	0.171*	
C33	0.1645 (2)	0.6617 (4)	0.56025 (12)	0.0669 (9)	
H33A	0.1087	0.6153	0.5709	0.080*	0.540 (12)
H33B	0.2126	0.5920	0.5563	0.080*	0.540 (12)
H33C	0.2243	0.6084	0.5614	0.080*	0.460 (12)
H33D	0.1146	0.6048	0.5719	0.080*	0.460 (12)
C34	0.2095 (12)	0.7431 (12)	0.6075 (4)	0.066 (3)	0.540 (12)
H34A	0.1596	0.8048	0.6163	0.079*	0.540 (12)
H34B	0.2616	0.7980	0.5971	0.079*	0.540 (12)
C35	0.2531 (8)	0.6640 (10)	0.6590 (3)	0.068 (3)	0.540 (12)
H35A	0.2014	0.6101	0.6704	0.082*	0.540 (12)
H35B	0.3035	0.6023	0.6509	0.082*	0.540 (12)
C36	0.2984 (9)	0.7568 (10)	0.7053 (4)	0.104 (4)	0.540 (12)
H36A	0.3532	0.8046	0.6952	0.156*	0.540 (12)
H36B	0.3216	0.7044	0.7383	0.156*	0.540 (12)
H36C	0.2494	0.8210	0.7121	0.156*	0.540 (12)
C35'	0.2274 (7)	0.7482 (16)	0.6599 (3)	0.084 (4)	0.460 (12)
H35C	0.2191	0.8236	0.6840	0.101*	0.460 (12)
H35D	0.1883	0.6727	0.6692	0.101*	0.460 (12)
C36'	0.3346 (8)	0.7077 (14)	0.6708 (7)	0.118 (5)	0.460 (12)
H36D	0.3423	0.6263	0.6505	0.177*	0.460 (12)
H36E	0.3570	0.6914	0.7098	0.177*	0.460 (12)
H36F	0.3733	0.7794	0.6591	0.177*	0.460 (12)
C34'	0.1879 (13)	0.7894 (15)	0.6001 (5)	0.073 (4)	0.460 (12)
H34C	0.2365	0.8465	0.5873	0.088*	0.460 (12)
H34D	0.1277	0.8424	0.5982	0.088*	0.460 (12)
F1	0.56013 (15)	0.8889 (3)	0.59104 (11)	0.1141 (9)	
N1	0.16778 (16)	0.3340 (3)	0.21066 (9)	0.0490 (6)	
N2	0.07372 (15)	0.5982 (2)	0.42205 (9)	0.0460 (6)	
N3	0.24634 (15)	0.6284 (2)	0.45583 (9)	0.0425 (5)	
N4	0.12771 (17)	0.7164 (3)	0.50414 (9)	0.0543 (6)	
O1	0.36369 (14)	0.5545 (2)	0.40787 (8)	0.0573 (6)	
S1	0.01216 (5)	0.46324 (8)	0.32512 (3)	0.0508 (2)	

### Atomic displacement parameters (Å^2^)

**Table:**

	*U*^11^	*U*^22^	*U*^33^	*U*^12^	*U*^13^	*U*^23^
C1	0.0445 (16)	0.074 (2)	0.0542 (19)	0.0018 (15)	0.0070 (14)	−0.0218 (17)
C2	0.079 (3)	0.107 (3)	0.085 (3)	−0.001 (2)	0.041 (2)	0.002 (2)
C3	0.087 (3)	0.182 (6)	0.097 (3)	−0.014 (4)	0.054 (3)	−0.018 (4)
C4	0.056 (3)	0.235 (8)	0.125 (5)	−0.007 (4)	0.039 (3)	−0.087 (5)
C5	0.061 (3)	0.156 (6)	0.174 (6)	0.032 (3)	0.016 (3)	−0.081 (5)
C6	0.067 (2)	0.089 (3)	0.112 (3)	0.014 (2)	0.008 (2)	−0.025 (2)
C7	0.0510 (16)	0.0564 (18)	0.0459 (17)	0.0009 (14)	0.0106 (13)	−0.0015 (14)
C8	0.0448 (15)	0.068 (2)	0.0388 (15)	0.0030 (14)	0.0046 (12)	−0.0073 (14)
C9	0.0400 (14)	0.0488 (16)	0.0347 (14)	−0.0002 (12)	0.0081 (11)	0.0020 (12)
C10	0.0399 (14)	0.0525 (17)	0.0355 (14)	−0.0058 (12)	0.0066 (11)	−0.0016 (12)
C11	0.058 (2)	0.119 (3)	0.055 (2)	−0.014 (2)	0.0159 (16)	−0.035 (2)
C12	0.0450 (15)	0.0556 (18)	0.0411 (16)	−0.0080 (13)	0.0071 (12)	−0.0064 (13)
C13	0.0488 (16)	0.067 (2)	0.0336 (15)	−0.0044 (14)	0.0038 (12)	−0.0170 (14)
C14	0.0482 (18)	0.077 (2)	0.066 (2)	−0.0068 (16)	0.0060 (15)	−0.0212 (18)
C15	0.053 (2)	0.120 (4)	0.094 (3)	−0.001 (2)	−0.002 (2)	−0.039 (3)
C16	0.084 (3)	0.113 (4)	0.068 (3)	0.033 (3)	−0.023 (2)	−0.029 (3)
C17	0.102 (3)	0.091 (3)	0.051 (2)	0.012 (2)	−0.012 (2)	−0.0048 (19)
C18	0.080 (2)	0.080 (3)	0.0490 (19)	−0.0041 (19)	−0.0023 (17)	0.0017 (18)
C19	0.0375 (14)	0.0448 (15)	0.0364 (14)	−0.0001 (11)	0.0058 (11)	0.0009 (12)
C20	0.0377 (14)	0.0516 (17)	0.0358 (14)	0.0007 (12)	0.0042 (11)	0.0012 (12)
C21	0.0390 (15)	0.0494 (17)	0.0355 (14)	0.0040 (12)	0.0051 (11)	0.0017 (12)
C22	0.0379 (14)	0.0559 (17)	0.0366 (15)	0.0056 (12)	0.0051 (11)	−0.0015 (13)
C23	0.0402 (14)	0.0524 (18)	0.0357 (14)	0.0009 (12)	0.0029 (11)	−0.0049 (12)
C24	0.0487 (16)	0.059 (2)	0.0503 (17)	0.0029 (14)	0.0008 (13)	0.0050 (15)
C25	0.064 (2)	0.061 (2)	0.085 (3)	−0.0114 (17)	0.0048 (18)	−0.0115 (19)
C26	0.0468 (18)	0.093 (3)	0.075 (2)	−0.0105 (18)	−0.0055 (16)	−0.026 (2)
C27	0.0550 (19)	0.083 (3)	0.065 (2)	0.0176 (17)	−0.0177 (16)	−0.0154 (19)
C28	0.0512 (17)	0.0601 (19)	0.0563 (19)	0.0085 (15)	−0.0060 (14)	−0.0049 (15)
C29	0.0628 (19)	0.058 (2)	0.0566 (19)	0.0073 (16)	0.0109 (15)	−0.0101 (16)
C30	0.062 (2)	0.069 (2)	0.071 (2)	0.0080 (17)	0.0174 (17)	−0.0034 (18)
C31	0.061 (2)	0.080 (2)	0.068 (2)	0.0180 (18)	0.0116 (17)	0.0058 (19)
C32	0.068 (3)	0.108 (4)	0.170 (5)	0.010 (2)	0.032 (3)	0.019 (3)
C33	0.0541 (18)	0.104 (3)	0.0436 (18)	0.0043 (18)	0.0125 (14)	−0.0012 (18)
C34	0.075 (8)	0.078 (6)	0.038 (5)	0.001 (5)	−0.003 (4)	0.002 (4)
C35	0.071 (6)	0.074 (6)	0.057 (4)	−0.016 (4)	0.006 (4)	−0.001 (4)
C36	0.123 (8)	0.112 (8)	0.068 (6)	−0.016 (6)	−0.004 (5)	−0.002 (5)
C35'	0.084 (7)	0.113 (11)	0.053 (6)	−0.016 (7)	0.005 (5)	−0.010 (6)
C36'	0.113 (10)	0.119 (10)	0.115 (12)	0.018 (8)	0.005 (8)	0.003 (8)
C34'	0.060 (7)	0.113 (11)	0.042 (6)	0.007 (7)	−0.003 (4)	−0.001 (7)
F1	0.0640 (13)	0.128 (2)	0.132 (2)	−0.0187 (13)	−0.0241 (13)	−0.0511 (17)
N1	0.0426 (13)	0.0682 (16)	0.0365 (12)	−0.0072 (11)	0.0083 (10)	−0.0095 (11)
N2	0.0389 (12)	0.0603 (15)	0.0380 (13)	0.0010 (11)	0.0055 (10)	−0.0082 (11)
N3	0.0376 (12)	0.0517 (14)	0.0365 (12)	0.0029 (10)	0.0028 (9)	−0.0032 (10)
N4	0.0461 (13)	0.0767 (18)	0.0388 (13)	0.0114 (12)	0.0047 (10)	−0.0105 (12)
O1	0.0381 (11)	0.0840 (15)	0.0489 (12)	0.0018 (10)	0.0061 (8)	−0.0122 (11)
S1	0.0361 (4)	0.0717 (5)	0.0441 (4)	−0.0058 (3)	0.0064 (3)	−0.0110 (4)

### Geometric parameters (Å, °)

**Table:**

C1—C2	1.373 (5)	C23—C28	1.382 (4)
C1—C6	1.385 (5)	C23—N3	1.452 (3)
C1—C7	1.509 (4)	C24—C25	1.380 (4)
C2—C3	1.378 (6)	C24—H24	0.9300
C2—H2	0.9300	C25—C26	1.366 (5)
C3—C4	1.345 (8)	C25—H25	0.9300
C3—H3	0.9300	C26—C27	1.352 (5)
C4—C5	1.343 (8)	C26—F1	1.363 (3)
C4—H4	0.9300	C27—C28	1.381 (4)
C5—C6	1.374 (7)	C27—H27	0.9300
C5—H5	0.9300	C28—H28	0.9300
C6—H6	0.9300	C29—C30	1.485 (4)
C7—N1	1.489 (3)	C29—N4	1.491 (4)
C7—C8	1.528 (4)	C29—H29A	0.9700
C7—H7	0.9800	C29—H29B	0.9700
C8—C9	1.488 (4)	C30—C31	1.521 (4)
C8—H8A	0.9700	C30—H30A	0.9700
C8—H8B	0.9700	C30—H30B	0.9700
C9—C10	1.355 (3)	C31—C32	1.491 (5)
C9—C19	1.439 (4)	C31—H31A	0.9700
C10—C12	1.501 (4)	C31—H31B	0.9700
C10—S1	1.741 (3)	C32—H32A	0.9600
C11—N1	1.468 (4)	C32—H32B	0.9600
C11—H11A	0.9600	C32—H32C	0.9600
C11—H11B	0.9600	C33—C34	1.444 (9)
C11—H11C	0.9600	C33—N4	1.473 (4)
C12—N1	1.472 (3)	C33—C34'	1.585 (12)
C12—C13	1.525 (4)	C33—H33A	0.9700
C12—H12	0.9800	C33—H33B	0.9700
C13—C14	1.374 (4)	C33—H33C	0.9700
C13—C18	1.385 (5)	C33—H33D	0.9700
C14—C15	1.401 (5)	C34—C35	1.506 (10)
C14—H14	0.9300	C34—H34A	0.9700
C15—C16	1.368 (6)	C34—H34B	0.9700
C15—H15	0.9300	C35—C36	1.494 (10)
C16—C17	1.379 (6)	C35—H35A	0.9700
C16—H16	0.9300	C35—H35B	0.9700
C17—C18	1.371 (5)	C36—H36A	0.9600
C17—H17	0.9300	C36—H36B	0.9600
C18—H18	0.9300	C36—H36C	0.9600
C19—C20	1.373 (3)	C35'—C36'	1.496 (12)
C19—C21	1.428 (3)	C35'—C34'	1.517 (11)
C20—N2	1.366 (3)	C35'—H35C	0.9700
C20—S1	1.734 (3)	C35'—H35D	0.9700
C21—O1	1.217 (3)	C36'—H36D	0.9600
C21—N3	1.424 (3)	C36'—H36E	0.9600
C22—N2	1.301 (3)	C36'—H36F	0.9600
C22—N4	1.375 (3)	C34'—H34C	0.9700
C22—N3	1.395 (3)	C34'—H34D	0.9700
C23—C24	1.366 (4)		
			
C2—C1—C6	117.6 (4)	C27—C26—F1	118.6 (3)
C2—C1—C7	121.9 (3)	C27—C26—C25	123.0 (3)
C6—C1—C7	120.5 (3)	F1—C26—C25	118.3 (4)
C1—C2—C3	120.4 (5)	C26—C27—C28	118.6 (3)
C1—C2—H2	119.8	C26—C27—H27	120.7
C3—C2—H2	119.8	C28—C27—H27	120.7
C4—C3—C2	121.4 (6)	C27—C28—C23	119.6 (3)
C4—C3—H3	119.3	C27—C28—H28	120.2
C2—C3—H3	119.3	C23—C28—H28	120.2
C5—C4—C3	118.9 (5)	C30—C29—N4	117.4 (3)
C5—C4—H4	120.5	C30—C29—H29A	108.0
C3—C4—H4	120.5	N4—C29—H29A	108.0
C4—C5—C6	121.4 (6)	C30—C29—H29B	108.0
C4—C5—H5	119.3	N4—C29—H29B	108.0
C6—C5—H5	119.3	H29A—C29—H29B	107.2
C5—C6—C1	120.3 (5)	C29—C30—C31	112.4 (3)
C5—C6—H6	119.9	C29—C30—H30A	109.1
C1—C6—H6	119.9	C31—C30—H30A	109.1
N1—C7—C1	111.3 (2)	C29—C30—H30B	109.1
N1—C7—C8	110.3 (2)	C31—C30—H30B	109.1
C1—C7—C8	110.6 (2)	H30A—C30—H30B	107.9
N1—C7—H7	108.2	C32—C31—C30	113.9 (3)
C1—C7—H7	108.2	C32—C31—H31A	108.8
C8—C7—H7	108.2	C30—C31—H31A	108.8
C9—C8—C7	111.5 (2)	C32—C31—H31B	108.8
C9—C8—H8A	109.3	C30—C31—H31B	108.8
C7—C8—H8A	109.3	H31A—C31—H31B	107.7
C9—C8—H8B	109.3	C31—C32—H32A	109.5
C7—C8—H8B	109.3	C31—C32—H32B	109.5
H8A—C8—H8B	108.0	H32A—C32—H32B	109.5
C10—C9—C19	112.0 (2)	C31—C32—H32C	109.5
C10—C9—C8	120.1 (2)	H32A—C32—H32C	109.5
C19—C9—C8	127.9 (2)	H32B—C32—H32C	109.5
C9—C10—C12	125.4 (2)	C34—C33—N4	124.2 (6)
C9—C10—S1	112.6 (2)	N4—C33—C34'	106.1 (6)
C12—C10—S1	121.97 (19)	C34—C33—H33A	106.3
N1—C11—H11A	109.5	N4—C33—H33A	106.3
N1—C11—H11B	109.5	C34—C33—H33B	106.3
H11A—C11—H11B	109.5	N4—C33—H33B	106.3
N1—C11—H11C	109.5	H33A—C33—H33B	106.4
H11A—C11—H11C	109.5	N4—C33—H33C	110.9
H11B—C11—H11C	109.5	C34'—C33—H33C	110.1
N1—C12—C10	109.2 (2)	N4—C33—H33D	110.8
N1—C12—C13	112.0 (2)	C34'—C33—H33D	110.2
C10—C12—C13	109.5 (2)	H33C—C33—H33D	108.8
N1—C12—H12	108.7	C33—C34—C35	115.1 (8)
C10—C12—H12	108.7	C33—C34—H34A	108.5
C13—C12—H12	108.7	C35—C34—H34A	108.5
C14—C13—C18	119.1 (3)	C33—C34—H34B	108.5
C14—C13—C12	119.9 (3)	C35—C34—H34B	108.5
C18—C13—C12	120.9 (3)	H34A—C34—H34B	107.5
C13—C14—C15	120.4 (4)	C36—C35—C34	111.1 (7)
C13—C14—H14	119.8	C36—C35—H35A	109.4
C15—C14—H14	119.8	C34—C35—H35A	109.4
C16—C15—C14	119.3 (4)	C36—C35—H35B	109.4
C16—C15—H15	120.3	C34—C35—H35B	109.4
C14—C15—H15	120.3	H35A—C35—H35B	108.0
C15—C16—C17	120.4 (4)	C36'—C35'—C34'	113.4 (10)
C15—C16—H16	119.8	C36'—C35'—H35C	108.9
C17—C16—H16	119.8	C34'—C35'—H35C	108.9
C18—C17—C16	120.1 (4)	C36'—C35'—H35D	108.9
C18—C17—H17	120.0	C34'—C35'—H35D	108.9
C16—C17—H17	120.0	H35C—C35'—H35D	107.7
C17—C18—C13	120.6 (4)	C35'—C36'—H36D	109.5
C17—C18—H18	119.7	C35'—C36'—H36E	109.5
C13—C18—H18	119.7	H36D—C36'—H36E	109.5
C20—C19—C21	118.1 (2)	C35'—C36'—H36F	109.5
C20—C19—C9	112.8 (2)	H36D—C36'—H36F	109.5
C21—C19—C9	129.0 (2)	H36E—C36'—H36F	109.5
N2—C20—C19	126.9 (2)	C35'—C34'—C33	112.1 (9)
N2—C20—S1	121.52 (19)	C35'—C34'—H34C	109.2
C19—C20—S1	111.6 (2)	C33—C34'—H34C	109.2
O1—C21—N3	120.2 (2)	C35'—C34'—H34D	109.2
O1—C21—C19	126.0 (2)	C33—C34'—H34D	109.2
N3—C21—C19	113.8 (2)	H34C—C34'—H34D	107.9
N2—C22—N4	120.1 (2)	C11—N1—C12	109.2 (2)
N2—C22—N3	123.0 (2)	C11—N1—C7	110.4 (2)
N4—C22—N3	116.8 (2)	C12—N1—C7	110.2 (2)
C24—C23—C28	120.5 (3)	C22—N2—C20	115.6 (2)
C24—C23—N3	120.0 (2)	C22—N3—C21	122.5 (2)
C28—C23—N3	119.4 (3)	C22—N3—C23	122.3 (2)
C23—C24—C25	120.0 (3)	C21—N3—C23	114.6 (2)
C23—C24—H24	120.0	C22—N4—C33	118.2 (2)
C25—C24—H24	120.0	C22—N4—C29	116.5 (2)
C26—C25—C24	118.3 (3)	C33—N4—C29	117.4 (2)
C26—C25—H25	120.9	C20—S1—C10	91.03 (12)
C24—C25—H25	120.9		
			
C6—C1—C2—C3	0.7 (6)	C24—C25—C26—F1	178.8 (3)
C7—C1—C2—C3	−178.7 (4)	F1—C26—C27—C28	−179.4 (3)
C1—C2—C3—C4	0.7 (7)	C25—C26—C27—C28	0.1 (6)
C2—C3—C4—C5	−1.0 (9)	C26—C27—C28—C23	−0.2 (5)
C3—C4—C5—C6	0.0 (9)	C24—C23—C28—C27	0.9 (5)
C4—C5—C6—C1	1.5 (8)	N3—C23—C28—C27	176.4 (3)
C2—C1—C6—C5	−1.8 (5)	N4—C29—C30—C31	178.4 (3)
C7—C1—C6—C5	177.7 (4)	C29—C30—C31—C32	178.4 (4)
C2—C1—C7—N1	56.1 (4)	N4—C33—C34—C35	−172.7 (8)
C6—C1—C7—N1	−123.3 (3)	C34'—C33—C34—C35	157 (5)
C2—C1—C7—C8	−66.9 (4)	C33—C34—C35—C36	179.6 (12)
C6—C1—C7—C8	113.7 (3)	C36'—C35'—C34'—C33	78.0 (18)
N1—C7—C8—C9	46.5 (3)	C34—C33—C34'—C35'	−25 (3)
C1—C7—C8—C9	170.1 (3)	N4—C33—C34'—C35'	−179.7 (10)
C7—C8—C9—C10	−14.1 (4)	C10—C12—N1—C11	172.2 (3)
C7—C8—C9—C19	166.6 (3)	C13—C12—N1—C11	−66.3 (3)
C19—C9—C10—C12	179.6 (2)	C10—C12—N1—C7	50.8 (3)
C8—C9—C10—C12	0.3 (4)	C13—C12—N1—C7	172.3 (2)
C19—C9—C10—S1	0.3 (3)	C1—C7—N1—C11	48.7 (3)
C8—C9—C10—S1	−179.0 (2)	C8—C7—N1—C11	171.9 (3)
C9—C10—C12—N1	−18.6 (4)	C1—C7—N1—C12	169.4 (2)
S1—C10—C12—N1	160.7 (2)	C8—C7—N1—C12	−67.5 (3)
C9—C10—C12—C13	−141.6 (3)	N4—C22—N2—C20	177.8 (2)
S1—C10—C12—C13	37.6 (3)	N3—C22—N2—C20	0.5 (4)
N1—C12—C13—C14	137.7 (3)	C19—C20—N2—C22	0.6 (4)
C10—C12—C13—C14	−100.9 (3)	S1—C20—N2—C22	179.5 (2)
N1—C12—C13—C18	−45.9 (4)	N2—C22—N3—C21	0.1 (4)
C10—C12—C13—C18	75.4 (3)	N4—C22—N3—C21	−177.3 (2)
C18—C13—C14—C15	−1.0 (5)	N2—C22—N3—C23	170.3 (3)
C12—C13—C14—C15	175.4 (3)	N4—C22—N3—C23	−7.1 (4)
C13—C14—C15—C16	0.3 (5)	O1—C21—N3—C22	176.8 (3)
C14—C15—C16—C17	0.6 (6)	C19—C21—N3—C22	−1.6 (4)
C15—C16—C17—C18	−0.9 (6)	O1—C21—N3—C23	5.9 (4)
C16—C17—C18—C13	0.2 (5)	C19—C21—N3—C23	−172.5 (2)
C14—C13—C18—C17	0.7 (5)	C24—C23—N3—C22	−64.1 (4)
C12—C13—C18—C17	−175.6 (3)	C28—C23—N3—C22	120.4 (3)
C10—C9—C19—C20	−0.8 (3)	C24—C23—N3—C21	106.9 (3)
C8—C9—C19—C20	178.5 (3)	C28—C23—N3—C21	−68.6 (3)
C10—C9—C19—C21	−178.4 (3)	N2—C22—N4—C33	119.2 (3)
C8—C9—C19—C21	0.9 (5)	N3—C22—N4—C33	−63.3 (4)
C21—C19—C20—N2	−2.2 (4)	N2—C22—N4—C29	−29.2 (4)
C9—C19—C20—N2	180.0 (3)	N3—C22—N4—C29	148.3 (3)
C21—C19—C20—S1	178.8 (2)	C34—C33—N4—C22	138.2 (9)
C9—C19—C20—S1	1.0 (3)	C34'—C33—N4—C22	148.7 (8)
C20—C19—C21—O1	−175.9 (3)	C34—C33—N4—C29	−73.7 (9)
C9—C19—C21—O1	1.5 (5)	C34'—C33—N4—C29	−63.2 (8)
C20—C19—C21—N3	2.5 (4)	C30—C29—N4—C22	85.0 (4)
C9—C19—C21—N3	179.9 (2)	C30—C29—N4—C33	−63.7 (4)
C28—C23—C24—C25	−1.5 (5)	N2—C20—S1—C10	−179.7 (2)
N3—C23—C24—C25	−177.0 (3)	C19—C20—S1—C10	−0.7 (2)
C23—C24—C25—C26	1.3 (5)	C9—C10—S1—C20	0.2 (2)
C24—C25—C26—C27	−0.6 (6)	C12—C10—S1—C20	−179.2 (2)

### Hydrogen-bond geometry (Å, °)

**Table:**

*D*—H···*A*	*D*—H	H···*A*	*D*···*A*	*D*—H···*A*
C4—H4···O1^i^	0.93	2.47	3.304 (6)	149

Symmetry codes: (i) −*x*+1, *y*−1/2, −*z*+1/2.
